# Poor Compliance to Clinical Guidelines in the Diagnosis of Acute Appendicitis: Insights from a National Survey

**DOI:** 10.3390/jcm13102862

**Published:** 2024-05-13

**Authors:** Nir Messer, Avi Benov, Adi Rov, Tali Bar-On, Oran Zlotnik, Jacob Chen, Haim Paran

**Affiliations:** 1 Sackler Faculty of Medicine, Tel Aviv University, Tel Aviv 6997801, Israel; adirov18@gmail.com (A.R.); talibaron07@gmail.com (T.B.-O.); oran.zlotnik@mail.mcgill.ca (O.Z.); jacov.hen@clalit.org.il (J.C.); paranh@clalit.org.il (H.P.); 2 Department of Surgery A, Meir Medical Center, Kfar Saba 4428164, Israel; 3 Israel Defense Forces, Medical Corps, Tel Hashomer, Ramat Gan 5262504, Israel; avi.benov@gmail.com; 4 The Azrieli Faculty of Medicine, Bar Ilan University, Safed 1311502, Israel; 5 Department of Surgery, Rabin Medical Center, Petach Tikva 4941492, Israel; 6 Hospital Administration, Meir Medical Center, Kfar Saba 4428164, Israel

**Keywords:** appendectomy, appendicitis, clinical guidelines, computed tomography

## Abstract

**Background**: Many scoring systems, algorithms, and guidelines have been developed to aid in the evaluation and diagnosis of acute appendicitis (AA). Many of these algorithms advocate against the routine use of radiological investigations when there is a high clinical suspicion of AA. However, there has been a significant rise in the use of imaging techniques for diagnosing AA in the past two decades. This is a national study aimed at assessing the adherence of residents assigned to the emergency department to the clinical guidelines for diagnosing AA. **Methods**: We introduced a case study of a male patient with highly suspicious clinical findings of AA to all surgical and emergency medicine residents assigned to the emergency department with the autonomy to make critical decisions to determine the preferred way of diagnosing AA. **Results**: A total of 62.4% of all relevant residents participated in this survey; 69.6% reported that the Alvarado score was eight or higher, and 82.1% estimated that the next step recommended by most clinical guidelines was appendectomy without further abdominal imaging tests. However, 83.4% chose to perform an imaging test to establish the diagnosis of AA. **Conclusions**: Our study revealed a notable non-adherence to clinical guidelines in diagnosing AA. Given the significance of these guidelines, we assert that adopting medical recommendations should not solely depend on individual education but should also be incorporated as a departmental policy.

## 1. Introduction

The diagnosis of acute appendicitis (AA) has undergone significant advancements in recent decades, incorporating factors such as patient history, symptoms, physical examination, and laboratory tests [1]. Nevertheless, the identification of AA remains intricate, given that approximately 55% of patients may lack the typical clinical signs and positive laboratory findings [2,3]. In the past, when the diagnosis relied solely on these data, the rates of unnecessary appendectomies reached as high as 15–25% [2].

Clinical practice guidelines are widely recognized as valuable instruments for enhancing the quality of healthcare [4]. In the context of acute appendicitis (AA), several scoring systems, algorithms, and guidelines have been developed to aid in evaluation and diagnosis, such as the Alvarado, Eskelinen, Ohman, Fenyo–Lindeberg, and Jerusalem guidelines, among others. Many of these algorithms advocate against the routine use of radiological investigations when there is a high clinical suspicion of AA, recommending instead that appendectomy be performed without further imaging. This approach has garnered widespread acceptance in surgical and emergency medicine textbooks [5,6,7].

Although clinical guidelines and scoring systems have proven effective, there has been a substantial increase in the utilization of imaging modalities such as computed tomography (CT), Ultrasonography (US), and Magnetic Resonance Imaging (MRI) for the diagnosis of appendicitis over the last two decades [8,9]. Regrettably, the current scarcity of data makes it difficult to determine the extent to which physicians adhere to these clinical guidelines. This study aimed to assess the adherence of residents assigned to the emergency department (ED) to the clinical guidelines for diagnosing acute appendicitis.

## 2. Methods

Following obtaining approval from the Institutional Review Board (IRB), 181 participants consisting of general surgeons (GS) and emergency medicine (EM) physicians were recruited for this study. This study focused on various aspects of AA diagnosis and treatment based on a case scenario of a male patient with a clear clinical diagnosis of AA. An extensive literature review did not identify a validated survey for assessing the diagnostic workup for AA diagnosis. To address this gap, the authors developed a questionnaire based on a thorough PubMed literature review using search terms related to AA diagnosis, adherence to guidelines, and imaging studies for AA in adults (Appendix A). The questionnaire was constructed on a web-based platform (Google Forms, Mountain View, CA, USA) and designed for individual self-completion. It consisted of three parts: Questions 1–5 gathered general professional information regarding medical and surgical experience, while questions 6–11 focused on evaluating a case of a male patient with a comprehensive medical history, symptoms, physical examination, and laboratory tests that indicated a clear clinical diagnosis of AA based on the existing literature and scoring systems. Question 12 allowed respondents to provide multiple responses regarding their motivations for choosing a preferred diagnostic workup. The questionnaire was reviewed by seven attending surgeons and two EM physicians for feedback and subsequently underwent a pilot involving twenty senior general surgeons and nineteen residents from surgical specialties parallel to general surgery. Following the pilot, the questionnaire was revised and approved.

Considering that in Israel, patient assessments are primarily carried out by residents during the morning shift and almost exclusively by residents during the afternoon and night shifts and recognizing that these residents possess the autonomy to make critical decisions concerning diagnosis, discharge, admission, or surgical procedures without requiring approval from attending staff, we intentionally recruited a homogeneous cohort of residents, excluding attending physicians, to mitigate potential biases. Following obtaining participants’ consent, the questionnaire was distributed through WhatsApp (WhatsApp Inc., Menlo Park, CA, USA) to all GS and EM residents (approximately 250 and 40, respectively) assigned to the emergency department who possessed the independence to make crucial decisions across all 19 university-affiliated hospitals in Israel. The participants were invited to complete the survey voluntarily and anonymously. An accompanying information message assured participants that their responses would be analyzed anonymously, and they had the option to skip any questions they preferred not to answer. As a result, not all questions were answered by all 181 respondents. The data collection period spanned 47 days, from 30 July 2017 to 14 September 2017. Reminder messages were sent five times during this period to encourage participation. Participants were divided into two subgroups: Postgraduate Year (PGY) ≤ 3 were classified as junior residents, while PGY ≥ 4 and above were considered senior residents.

This study’s primary outcome was to assess the adherence of general surgeons and EM residents to clinical guidelines for diagnosing acute appendicitis (AA). This evaluation was conducted by analyzing the actual diagnostic workup employed by participants and determining their level of awareness regarding the current guidelines for AA diagnosis. Additionally, this study aimed to identify the factors that influenced participants in selecting their preferred diagnostic workup for AA.

All statistical analyses were performed using SPSS, version 25 (IBM Corp., Armonk, NY, USA). Data are presented as numbers and percentages. Fisher’s exact or chi-square tests were used in univariate analyses. A *p*-value less than 0.05 was considered statistically significant.

## 3. Results

A total of 181 residents completed the questionnaire, resulting in a response rate of 62.4%. Among the respondents, 161 were GS residents, accounting for 64.4% of all relevant Israeli GS residents. A total of 21 participants were EM residents, accounting for 50% of all relevant Israeli EM residents. A total of 83 (45.9%) participants were categorized as junior residents, while 98 (54.1%) were classified as senior residents. A total of 118 general surgery residents (73.7%) reported performing more than 30 appendectomies independently. Regarding waiting times for diagnostic tests, 152 (84%) reported a waiting time of shorter than 2 h for CT scans, while 149 (82.3%) reported a waiting time of shorter than 2 h for US tests (Table 1).

The diagnostic workout is outlined in Table 2. The mean Alvarado score is 7.9 (SD = 1.6), with 126 (69.6%) participants estimating that the Alvarado score is 8 or higher. Following reviewing the presented case, 147 (81.2%) participants presumed that the next step recommended by the guidelines was an appendectomy without further abdominal imaging tests, while 34 (18.8%) opted for abdominal imaging tests. When asked “Depending on your experience, what is the next step?”, 151 (83.4%) participants chose to perform an abdominal imaging test, while 30 (16.6%) decided to have an appendectomy without an imaging test. Among the 147 (81.2%) participants who chose an appendectomy as per the guidelines, 119 (80.9%) decided to perform an imaging test as the next step based on their personal experience. Among the participants who chose imaging tests, 111 (61.3%) selected abdominal ultrasound and 40 (22.1%) opted for a CT scan as the next step. Regarding the question “in case you choose the abdominal US as the first step, what will be your next step after a non-diagnostic ultrasound”, 103 participants (79.8%) chose to proceed with a CT scan, while 26 (20.2%) opted for an appendectomy. When asked about the reasons for selecting an imaging test before surgery, 96 (53%) participants mentioned that it was the common practice in their department, 69 (38.1%) chose imaging tests to “strengthen” the diagnosis, and 55 (30.4%) considered the patient’s expectation for a specific diagnosis.

Table 3 outlines a comparison by seniority. No significant difference was seen regarding the Alvarado score, with 57 junior residents (85.1%) and 69 senior residents (78.4%) estimating that the Alvarado score is 8 or higher (*p* = 0.292). A total of 65 junior residents (78.3%) and 82 senior residents (83.7%) presumed that the next step recommended by the guidelines was an appendectomy without further abdominal imaging tests (*p* = 0. 461). Regarding the question “Depending on your experience, what is the next step?”, 75 junior residents (90.4%) and 76 senior residents (77.6%) chose the abdominal imaging test (*p* = 0.21). Among them, 60 junior residents (72.3%) and 51 senior residents (52.1%) chose US, while 15 junior residents (18.1%) and 25 senior residents (25.5%) chose the CT scan as the first test (*p* = 0.013). Regarding the question “In case you choose the abdominal US as the first step, what will be your next step after a non-diagnostic US?”, fifty-six junior residents (88.9%) and forty-seven senior residents (71.2%) chose CT, while seven (11.1%) junior residents and nineteen senior residents (28.8%) chose the appendectomy (*p* = 0.012). A comparison between GS and EM residents regarding the diagnostic workup of AA is presented in Supplementary Table S1.

## 4. Discussion

Our study indicates a lack of adherence to clinical guidelines in diagnosing AA, as the majority of residents rely on imaging tests, in contrast to the guidelines’ recommendations. Despite the participants’ overall understanding that the presented case was a clear case of AA that warrants an appendectomy without needing imaging tests, the majority still chose to proceed with such tests. This pattern was consistent across most study participants, and neither seniority nor clinical experience substantially influenced their preference for imaging tests.

To the best of our knowledge, this study constitutes the most comprehensive survey dedicated to examining the diagnosis and adherence to clinical guidelines in AA. This analysis sheds light on a discrepancy between the recommendations provided by the guidelines and the practical diagnostic approach. It can be hypothesized that residents’ tendency to perform additional imaging tests may stem from the inherent challenges associated with clinically diagnosing AA. Historically, when clinical signs were the primary diagnostic basis, negative appendectomy rates were alarmingly high, ranging from 15% to 25% [8,9]. Furthermore, the morbidity associated with “negative appendectomy” surgery was comparable with an uncomplicated appendectomy [10]. As a result, radiological imaging tests have been pursued to enhance preoperative diagnostic accuracy [2]. Remarkably, computed tomography has demonstrated a sensitivity of 98.5% and a specificity of 98% in diagnosing AA [11,12]. Consequently, the availability of improved radiological imaging tests has substantially reduced negative appendectomy rates to 5% [13,14].

Nevertheless, our investigation reveals that additional factors motivate residents to opt for these additional tests, despite knowing that clinical guidelines do not necessitate them. Notably, 30% of participants cited the patient’s expectation of a definitive diagnosis as a key factor in their decision to opt for imaging tests. Furthermore, 25.9% expressed concerns about potential medical malpractice, which led them to select imaging tests as a precautionary measure. Interestingly, the prevailing “departmental common practice” emerged as the strongest motivating factor, accounting for 53% of the participants’ choices. Intriguingly, our comprehensive review of the existing literature uncovered evidence suggesting that the influence of the peer group can sometimes surpass the impact of recommendations found in the established academic literature [15]. This notable phenomenon calls for further research to comprehend its implications fully.

Another notable instance of non-adherence to clinical guidelines is the choice of imaging tests for diagnosing acute appendicitis (AA). In most clinical guidelines, especially in borderline cases, a CT scan is recommended [5,6,7]. However, our findings reveal that a significant number of participants opted for an abdominal US, despite its limited sensitivity (70%) [16]. This observation becomes even more intriguing considering the substantial increase in the utilization of CT scans in EM compared to other imaging tests over the past decade [17]. One possible rationale for this preference towards ultrasound might be its radiation-free nature. Nevertheless, further investigation is warranted to better understand the underlying factors contributing to this choice.

A comprehensive literature review was conducted to examine whether the lack of adherence to clinical guidelines in diagnosing AA is an isolated occurrence or indicative of a broader trend. The review revealed that the discrepancy in physician adherence to guidelines is more prevalent than initially anticipated, as supported by several studies [1,3,18]. Although the exact reasons for this misalignment are not fully elucidated, several theoretical explanations have been proposed. Cabana et al. conducted a review of 33 surveys and identified specific barriers to the adoption of guideline recommendations [19], including a lack of agreement with a specific guideline. Cabana et al. found that 10% of respondents disagreed with guidelines for various reasons. These included guidelines being based on incorrect interpretations of evidence or perceiving that the benefits of the recommendations did not outweigh patient risks. In our study, 38% of participants stated that the reason for performing an imaging study was to “strengthen” the diagnostic findings before surgery, which may reflect the belief that guideline recommendations did not adequately justify the risks involved. Secondly, the inertia of previous practices emerged as a significant barrier hindering the adoption of new guidelines. Physicians often find it challenging to overcome the established practices and may lack the motivation to change. In our study, 53% of participants cited “departmental common practice” as a key factor influencing their decision to perform imaging tests. We speculate that the influence of the peer group may sometimes outweigh the ability to embrace alternative recommendations. Additionally, patient-related barriers were highlighted as another obstacle to adherence. In Cabana et al.’s review, 10% of respondents indicated that patient preferences contradicting guideline recommendations posed a challenge. In our study, 30% of participants cited the patient’s expectation of a definitive diagnosis as a significant factor in opting for imaging tests. This may reflect patients’ desire for a diagnosis based on more advanced or sophisticated tests before undergoing surgery. Overall, our literature review demonstrates that the observed lack of adherence to clinical guidelines in diagnosing AA is not an isolated case but represents a broader trend. This trend is influenced not only by medical factors but also by social influences, including the attitudes of physicians, departmental practices, and patient expectations.

This study is subject to several limitations that should be taken into consideration. Firstly, the decision to operate was assessed through a questionnaire rather than a prospective study, which introduces potential confounders due to possible differences between the subjects in each group and the presence of reporting bias. However, we chose this approach to efficiently address the research questions while avoiding ethical concerns associated with interfering with clinical decisions. Additionally, criticism may be raised concerning this study’s design, advocating for the inclusion of multiple case studies and more challenging cases to encompass greater variability. However, we recognized that such added complexity would deviate from our primary objective, which focused on assessing adherence to guidelines rather than evaluating the diagnostic methods for AA. Thus, we selected a straightforward case that minimized ambiguity to ensure clarity in outlining adherence to guidelines. Moreover, this study relies on various guidelines and scoring systems, which obviate the necessity of performing CT scans in cases highly suggestive of AA. Alternatively, other scoring systems, such as the Jerusalem guidelines, advocate for the use of CT scans to establish definitive diagnoses [20,21,22]. However, it is noteworthy that even within the Jerusalem guidelines, in high-risk patients under 40 years of age (with an AIR score of 9–12, an Alvarado score of 9–10, and an AAS ≥ 16), CT scans may be omitted prior to diagnostic ± therapeutic laparoscopy. Furthermore, other unaccounted factors might have influenced the results, such as the variations between different hospitals and the individual experiences of the participants. Individual experience, in particular, could have impacted the decision to proceed with appendectomy without imaging. Thirdly, the competence level of the participants was measured quantitatively based on their seniority and the number of appendectomies performed. However, these parameters may not necessarily correlate with actual competency and expertise in diagnosing AA. Another limitation is that the number of emergency physicians included in this study was relatively small (42%), although the overall response rates were high (51.7% of general surgery and EM residents in Israel). Lastly, the availability of imaging modalities, such as CT and US, in various centers is an important consideration. While our study assumed the widespread availability of these modalities in Western hospitals, it is essential to acknowledge that some centers may lack these facilities. Consequently, our findings may not be applicable in such settings.

## 5. Conclusions

The findings of our study shed light on the notable non-adherence to clinical guidelines in the diagnosis of AA. The adoption of clinical guideline recommendations is vital for establishing standardized diagnostic protocols and treatment strategies, as well as for guiding trainee education. Given the significance of these guidelines, we assert that the adoption of medical recommendations should not solely depend on individual education but should also be incorporated as a departmental policy. By emphasizing the importance of a collective approach to guideline adherence, we can effectively promote the implementation of evidence-based practices, enhance diagnostic accuracy, optimize treatment outcomes, and improve the education and training of medical professionals.

## Figures and Tables

**Table 1:** Demographics and hospital characteristics.

Demographics and Hospital Characteristics	*n* = 181	
Profession	General surgery	161 (88.9%)
	Emergency medicine	20 (11.1%)
Seniority	Junior residents	83 (45.8%)
	Senior residents	98 (54.1%)
Surgical experience ^1^	<50	62 (38.5%)
	>50	99 (61.5%)
US availability (hours)	<2	149 (82.3%)
	>2	32 (17.7%)
CT availability (hours)	<2	152 (84%)
	>2	29 (16%)

^1^ Surgical experience by number of appendectomies.

**Table 2:** Diagnostic workout.

Question	Answer	*n* = 181
What is the Alvarado score in this case?	<8	29 (16%)
	≥8	126 (69.6.3%)
What is the next step recommended by the guidelines?	Appendectomy	147 (81.2%)
	Abdominal US	13 (7.2%)
	Abdominal CT	21 (11.6%)
	Abdominal MRI	0
What is the next step recommended by the guidelines?	Appendectomy	147 (81.2%)
	Abdominal imaging test	34 (18.8%)
Based on your experience, what is the next step?	Appendectomy	30 (16.6%)
	Abdominal US	111 (61.3%)
	Abdominal CT	40 (22.1%)
	Abdominal MRI	0
Based on your experience, what is the next step?	Appendectomy	30 (16.6%)
	Abdominal imaging test	151 (83.4%)
In case you choose the abdominal US as the first step, what will be your next step after a non-diagnostic ultrasound?	Appendectomy	26 (20.2%)
	Abdominal CT	103 (79.8%)
	Abdominal MRI	0
If you chose an imagining scan as the first step, what is the reason?	Literature recommendation	5 (2.8%)
	Common practice in my department	96 (53.0%)
	Anamnesis and physical examination were equivocal	16 (8.8%)
	Needed to “strengthen” the diagnostic findings before surgery	69 (38.1%)
	Fear of medical malpractice	47 (26.0%)
	The patient’s expectation of a definite diagnosis	55 (30.4%)

**Table 3:** Comparison by seniority.

Question		Total Cohort	Junior Residents	Senior Residents	*p*-Value
		*n* = 181	*n* = 83	*n* = 98	
What is the Alvarado score in this case?	<8	29 (18.7%)	10 (14.9%)	19 (21.6%)	0.29
	≥8	126 (81.3%)	57 (85.1%)	69 (78.4%)	
What is the next step recommended by the guidelines?	Appendectomy	147 (81.2%)	65 (78.3%)	82 (83.7%)	0.65
	Abdominal ultrasound	13 (7.2%)	7 (8.4%)	6 (6.1%)	
	Abdominal CT	21 (11.6%)	11 (13.2%)	10 (10.2%)	
	Abdominal MRI	0	0	0	
What is the next step recommended by the guidelines?	Appendectomy	147 (81.2%)	65 (78.3%)	82 (83.6%)	0.46
	Abdominal imaging test	34 (18.8%)	18 (21.6%)	16 (16.3%)	
Based on your experience, what is the next step?	Appendectomy	30 (16.6%)	8 (9.6%)	22 (22.4%)	0.013
	Abdominal ultrasound	111 (61.3%)	60 (72.3%)	51 (52.1%)	
	Abdominal CT	40 (22.1%)	15 (18.1%)	25 (25.5%)	
	Abdominal MRI	0	0	0	
Based on your experience, what is the next step?	Appendectomy	30 (16.6%)	8 (9.6%)	22 (22.4%)	0.21
	Abdominal imaging test	151 (83.4%)	75 (90.4%)	76 (77.6%)	
In case you choose the abdominal US as the first step, what will be your next step after a non-diagnostic ultrasound?	Appendectomy	26 (20.2%)	7 (11.1%)	19 (28.8%)	0.01
	Abdominal CT	103 (79.8%)	56 (88.9%)	47 (71.2%)	
	Abdominal MRI	0	0	0	
If you chose an imagining scan as the first step, what is the reason?	Literature recommendation	5 (2.8%)	3 (3.6%)	2 (2.0%)	0.66
	Common practice in my department	96 (53.0%)	43 (51.8%)	53 (54.1%)	0.76
	Anamnesis and physical examination were equivocal	16 (8.8%)	8 (9.6%)	8 (8.2%)	0.73
	Needed to “strengthen” the diagnostic findings before surgery	69 (38.1%)	37 (44.6%)	32 (32.7%)	0.1
	Fear of medical malpractice	47 (26.0%)	20 (24.1%)	27 (27.6%)	0.6
	Patient’s expectation of a definite diagnosis	55 (30.4%)	18 (21.7%)	37 (37.8%)	0.02

## Data Availability

The data presented in this study are available on request from the corresponding author. The data are not publicly available due to privacy.

## Supplementary Materials

The following supporting information can be downloaded at: https://www.mdpi.com/article/10.3390/jcm13102862/s1, Table S1. Comparison between general surgery and emergency medicine residents.

## Appendix A

**Questionnaire**

1. Hospital Name
2. Residency: General Surgery or Emergency Medicine
3. Post Graduate Year—General Surgery resident: 2, 3, 4, 5, 6
4. Seniority at the Emergency Medicine department (including general surgery residents): 2, 3, 4.
5. Number of appendectomies performed: less than 10, 10–30, 30–50, 50–100, more than 100.

**Case Study**

A 25 year old male suffering since yesterday from periumbilical abdominal pain that extended to his right lower quadrant. He also suffered from anorexia and vomited twice. On admission to the emergency department, his pulse was 90 bpm, blood pressure 120/80 mmHg, saturation 99% (room air), temperature 37.8 °C, BMI 24. On physical examination, he has significant right lower quadrant tenderness, rebound, and Rovsing’s sign is positive. Lab results: WBC 12,000, Neutrophilia 1000, CRP 3.

1. Based on your experience, what is the next step? (Appendectomy, abdominal ultrasound, abdominal CT, abdominal MRI).
2. If you chose an abdominal ultrasound, the test results are that the appendix was not visualized. What is your next step? (Appendectomy, abdominal CT, abdominal MRI).
3. What is the Alvarado score in this case?
4. What is the availability of abdominal US in your hospital? (one hour, two hours, two hours to four hours, more than four hours).
5. What is the availability of abdominal CT in your hospital? (one hour, two hours, two hours to four hours, more than four hours).
6. What is the next recommended step by the guidelines? (Appendectomy, abdominal ultrasound, abdominal CT, abdominal MRI).
7. If you chose to have an imaging scan as the first step, what is the reason for that? Literature recommendation, common practice in my department, anamnesis and physical examination are equivocal, need to “strengthen” the diagnostic findings before surgery, fear of medical malpractice, the patient expects a definite diagnosis, other.

## References

[1] Gorter R.R., Eker H.H., Gorter-Stam M.A.W., Abis G.S.A., Acharya A., Ankersmit M., Antoniou S.A., Arolfo S., Babic B., Boni L. (2016). Diagnosis and management of acute appendicitis. EAES consensus development conference 2015. Surg. Endosc..

[2] Kabir S.A., Kabir S.I., Sun R., Jafferbhoy S., Karim A. (2017). How to diagnose an acutely inflamed appendix; a systematic review of the latest evidence. Int. J. Surg..

[3] Bakker O.J., Go P.M., Puylaert J.B., Kazemier G., Heij H.A., Werkgroup en Klankbordgroup “Richtlijn Acute Appendicitis” (2010). Guideline on diagnosis and treatment of acute appendicitis: Imaging prior to appendectomy is recommended. Ned. Tijdschr. Geneeskd..

[4] Grimshaw J., Thomas R., MacLennan G., Fraser C., Ramsay C., Vale L., Whitty P., Eccles M., Matowe L., Shirran L. (2004). Effectiveness and efficiency of guideline dissemination and implementation strategies. Health Technol. Assess..

[5] (2022). Sabiston Textbook of Surgery.

[6] Schwartz S.I., Brunicardi F.C. (2010). Schwartz’s Principles of Surgery.

[7] Stapczynski J.S., Tintinalli J.E. (2011). Tintinalli’s Emergency Medicine: A Comprehensive Study Guide.

[8] McCallion J., Canning G.P., Knight P.V., McCallion J.S. (1987). Acute appendicitis in the elderly: A 5-year retrospective study. Age Ageing.

[9] Rothrock S.G., Green S.M., Dobson M., Colucciello S.A., Simmons C.M. (1995). Misdiagnosis of appendicitis in nonpregnant women of childbearing age. J. Emerg. Med..

[10] Allaway M.G.R., Eslick G.D., Cox M.R. (2019). The Unacceptable Morbidity of Negative Laparoscopic Appendicectomy. World J. Surg..

[11] Balthazar E.J., Megibow A.J., Siegel S.E., Birnbaum B.A. (1991). Appendicitis: Prospective evaluation with high-resolution CT. Radiology.

[12] Balthazar E.J., Birnbaum B.A., Yee J., Megibow A.J., Roshkow J., Gray C. (1994). Acute appendicitis: CT and US correlation in 100 patients. Radiology.

[13] Raja A.S., Wright C., Sodickson A.D., Zane R.D., Schiff G.D., Hanson R., Baeyens P.F., Khorasani R. (2010). Negative appendectomy rate in the era of CT: An 18-year perspective. Radiology.

[14] Flum D.R., McClure T.D., Morris A., Koepsell T. (2005). Misdiagnosis of appendicitis and the use of diagnostic imaging. J. Am. Coll. Surg..

[15] Grimshaw J., Freemantle N., Wallace S., Russell I., Hurwitz B., Watt I., Long A., Sheldon T. (1995). Developing and implementing clinical practice guidelines. Qual. Health Care.

[16] Kamiński M. (2018). Does abdominal ultrasound is a useful tool in appendicitis diagnosis?. Pol. Przegl. Chir..

[17] Kocher K.E., Meurer W.J., Fazel R., Scott P.A., Krumholz H.M., Nallamothu B.K. (2011). National trends in use of computed tomography in the emergency department. Ann. Emerg. Med..

[18] Viniol A., Keunecke C., Biroga T., Stadje R., Dornieden K., Bösner S., Donner-Banzhoff N., Haasenritter J., Becker A. (2014). Studies of the symptom abdominal pain—A systematic review and meta-analysis. Fam. Pract..

[19] Cabana M.D., Rand C.S., Powe N.R., Wu A.W., Wilson M.H., Abboud P.-A.C., Rubin H.R. (1999). Why don’t physicians follow clinical practice guidelines? A framework for improvement. JAMA.

[20] Bass G.A., Mohseni S., Ryan J., Forssten M.P., Tolonen M., Cao Y., Kaplan L.J., Hulme R.A., Biloslavo A., Kurihara H. (2023). Clinical practice selectively follows acute appendicitis guidelines. Eur. J. Trauma Emerg. Surg..

[21] Di Saverio S., Podda M., De Simone B., Ceresoli M., Augustin G., Gori A., Boermeester M., Sartelli M., Coccolini F., Tarasconi A. (2020). Diagnosis and treatment of acute appendicitis: 2020 update of the WSES Jerusalem guidelines. World J. Emerg. Surg..

[22] Fugazzola P., Ceresoli M., Agnoletti V., Agresta F., Amato B., Carcoforo P., Catena F., Chiara O., Chiarugi M., Cobianchi L. (2020). The SIFIPAC/WSES/SICG/SIMEU guidelines for diagnosis and treatment of acute appendicitis in the elderly (2019 edition). World J. Emerg. Surg..

