# Supplementary material for: Poor Compliance to Clinical Guidelines in the Diagnosis of Acute Appendicitis: Insights from a National Survey
Source: J Clin Med. 2024 May 13;13(10):2862. doi: 10.3390/jcm13102862 (PMC11122132; doi:10.3390/jcm13102862)
Supplement: Supplementary file 1 [file jcm-13-02862-s001.zip › jcm-2990576-supplementary.pdf]

Journal of Clinical Medicine

MDPI

Table S1. Comparison between general surgery and emergency medicine residents.

Question	Total Cohort (n = 181)	General Surgery Resident (n = 161)	Emergency Medicine Residents (n = 20)	*p-value*
What is the Alvarado score in this case?				
Alvarado scores up to 7	29 (16%)	26 (19%)	3 (16.7%)	0.999
Alvarado above 7	126 (69.6.3%)	111 (81%)	15 (83.3%)	
What is the next recommended step in the guidelines?				
appendectomy	147 (81.2%)	135 (84.4%)	12 (57.1%)	
abdominal ultrasound	13 (7.2%)	9 (5.6%)	5 (19%)	
abdominal CT	21 (11.6%)	16 (10%)	5 (23.8%)	
abdominal MRI	0	0	0	
What is the next recommended step in the guidelines?				0.003
appendectomy	147 (81.2%)	135 (84.4%)	12 (57.1%)	
abdominal imaging test	34 (18.8%)	25 (15.6%)	9 (52.9%)	
Based on your experience, what is the next step?				
appendectomy	30 (16.6%)	28 (17.5%)	2 (9.5%)	
abdominal ultrasound	111 (61.3%)	94 (58.8%)	17 (81%)	
abdominal CT	40 (22.1%)	38 (23.8%)	2 (9.5%)	
abdominal MRI	0	0	0	
Based on your experience, what is the next step?				
appendectomy	30 (16.6%)	28 (17.5%)	2 (9.5%)	0.535
abdominal imaging test	151 (83.4%)	132 (82.5%)	19 (90.5%)	
In case you chose an abdominal ultrasound, the test results are that the appendix wasn't visualized. What is your next step?				0.525
Appendectomy	26 (20.2%)	24 (21.6%)	2 (11.1%)	
abdominal CT	103 (79.8%)	87 (78.4%)	16 (88.9%)	
abdominal MRI	0	0	0	
In case you chose to have an imagining scan as first step, what is the reason for that?				
literature recommendation	5 (2.8%)	4 (2.5%)	1 (4.8%)	0.55
common practice in my department	96 (53%)	87 (54.4%)	9 (42.9%)	0.32
anamnesis and physical examination are not unequivocal	16 (8.8%)	11 (6.9%)	5 (23.8%)	0.01
necessity to "strengthen" the diagnostic findings before surgery	69 (38.1%)	56 (35%)	13 (61.9%)	0.017
fear of medical malpractice	47 (26%)	42 (26.3%)	5 (23.8%)	0.81
patient expectation of a definite diagnosis	55 (30.4%)	53 (33.1%)	2 (9.5%)	0.027
